# The semi-extended infrapatellar intramedullary nailing of distal tibia fractures: a randomized clinical trial

**DOI:** 10.1186/s10195-022-00674-3

**Published:** 2022-11-28

**Authors:** Ke Lu, Zhi-qiang Wu, Hong-zhen Wang, Rong-xun Qian, Chong Li, Yi-jun Gao

**Affiliations:** Department of Orthopaedics, Affiliated Kunshan Hospital of Jiangsu University, No. 91 West of Qianjin Road, Suzhou, 215300 Jiangsu China

**Keywords:** Semi-extended, Intramedullary nailing, Suprapatellar, Parapatellar, Infrapatellar, Distal tibial fracture, Malalignment

## Abstract

**Background:**

Malalignment is a common event during the intramedullary nailing (IMN) of distal tibia fractures (DTFs). Although it is reported that the semi-extended IMN techniques such as suprapatellar (SP) and parapatellar (PP) approaches may be superior in preventing malalignment, the application of these techniques is concerning owing to the intra-articular involvement. We thus developed an extra-articular semi-extended infrapatellar (SEIP) approach which utilizes the infrapatellar (IP) space while maintaining the knee in a semi-extended position. However, there are no studies on the safety and efficacy of SEIP in treating DTFs. Therefore, in this study, the SEIP technique was examined, particularly in terms of the potential alignment improvement of DTFs, and this technique was compared with the traditional hyperflexed infrapatellar (HFIP) procedure.

**Materials and methods:**

This randomized clinical trial (RCT) compared IMN malalignment while correcting extraarticular and nondisplaced intra-articular DTFs between April 2018 and June 2021 using the HFIP and SEIP techniques at a level I trauma center in China. The study participants were clinically and radiographically examined for at least 12 months of follow-ups. Intraoperative fluoroscopy time, operation time, blood loss, hospitalization duration, functional ankle score, and complications were assessed as well.

**Results:**

Among the 88 recruited participants, 45 (51%) underwent traditional HFIP IMN and 43 (49%) underwent SEIP IMN. Malalignment occurred in 9 patients (20.0%) from the HFIP cohort and in 2 patients (4.7%) from the SEIP cohort (*P* value = 0.030). In addition, the SEIP IMN technique significantly reduced the intraoperative fluoroscopy time, operation time, and improved the postoperative ankle function compared to the HFIP IMN technique. However, the intraoperative blood loss, hospitalization duration, infection, delay union, and nonunion remained the same between the two cohorts.

**Conclusions:**

In summary, we demonstrated that the SEIP IMN provides markedly enhanced alignment of extraarticular and nondisplaced intra-articular DTFs compared to the traditional HFIP IMN procedure. The described technique represents an effective option for IMN of DTFs.

**Level of evidence:**

Level 2.

***Trial registration*** The Chinese Clinical Trial Registry, ChiCTR2100043673. Registered 26 February 2021, retrospectively registered, http://www.chictr.org.cn/showprojen.aspx?proj=122263

## Introduction

Distal tibial fractures (DTFs) are the leading fractures involving the lower extremity, and they account for 10–13% of all tibial fractures [1]. Unfortunately, treating DTFs remains a challenge to this day. An inadequate soft-tissue envelope, adjoining ankle joint, and underlying trauma mechanisms induce complications following fractures [2]. Given these challenges, minimally invasive treatment procedures such as minimally invasive plating (MIPO) and intramedullary nailing (IMN) are preferred, despite certain drawbacks. Multiple reports suggest that MIPO is accompanied by an elevated rate of tissue breakdown, infection, and implant-associated complications [3, 4]. In contrast, IMN is a minimally invasive fixation process that prevents additional soft-tissue damage by promoting endogenous osteosynthesis. Nevertheless, IMN has a tendency towards malalignment [5–8]. It is yet unknown whether this is due to the inability to maintain reduction during IMN, especially during moments of flexion or extension, or due to a reduction loss owing to the large distal tibia diameter and resulting loss of interference fit with the nail [8].

Traditional tibial nailing is done through entry portal placement via a hyper-flexed infrapatellar (HFIP) approach, whereby either a patellar tendon-splitting or patellar tendon-sparing method is used while the knee remains in hyperflexion. During canal preparation, knee flexion and extension are required for fluoroscopic image-based visualization of the instrument and implant placements. In contrast, the suprapatellar (SP) and parapatellar (PP) IMN techniques forgo extremity manipulations, as the limb is placed in a semi-extended condition during the entire operation. Hence, a DTF can be reduced and persistently maintained during medullary canal preparation and IMN placement [8]. However, the application of these techniques is concerning owing to their intra-articular nature, as they can have complications such as knee cartilage damage [9, 10], septic arthritis [11], and heterotopic ossification [12]. In addition, the application of the SP IMN technique requires special surgical instruments.

Recently, we developed an extra-articular semi-extended infrapatellar (SEIP) approach that utilizes the infrapatellar (IP) space while the knee in a semi-extended position [13]. The aforementioned retrospective study demonstrated that, when treating tibial shaft fractures, the SEIP technique can markedly reduce the intra-surgical fluoroscopy duration, surgical duration, and knee pain and can enhance postsurgical knee function relative to the traditional HFIP technique [13]. However, to date, there are no studies on the safety and efficacy of the SEIP technique in treating DTFs. Thus, the current investigation assessed the performance of the SEIP approach in enhancing DTF alignment relative to the classical HFIP procedure. We speculated that the SEIP approach would enhance DTF alignment in patients treated with IMN. We also compared the intraoperative indicators, foot and ankle functions, and associated complications.

### Patients and methods

This randomized clinical trial (RCT) was conducted in a level I trauma center in China. We received ethical approval from the Affiliated Kunshan Hospital of Jiangsu University (approval no. 2020-04-024-K01) and strictly followed the guidelines of the Declaration of Helsinki. All participants provided informed consent prior to the initiation of the study. This work is registered in the Chinese Clinical Trial Registry (ChiCTR2100043673). This study selected adult participants over 18 years of age with an acute closed or Gustilo I DTF occurring between April 2018 and June 2021 who received their final follow-up in July 2022. DTFs were classified based on the Orthopaedic Trauma Association (OTA) stratification system using initial injury films and computed tomography (CT). The following patients were selected for analysis: those with extraarticular fractures (OTA 43-A) and (OTA 43-C1 and C2) with a nondisplaced intraarticular fracture line [8]. Moreover, we only included fractures with major fracture lines located within 12 cm of the distal tibial plafond [14]. The following patients were eliminated from the analysis: those with a fracture occurring too distal to achieve proper fixation of four cortices using distal interlocking screws [5] and those with an ipsilateral proximal tibia fracture or knee injury, prior knee or ankle surgery preexisting ankle arthrodesis, and pathological fracture. In addition, we also eliminated patients with open Gustilo II or III fractures or fractures with a displaced intraarticular fragment. Following consent, the participants were arbitrarily separated into two populations in a 1:1 ratio: those receiving the HFIP IMN (control) or the SEIP IMN (experimental) treatment. The IMN (Tibia Without X-ray-Excellent [TWX-E] instrument system; Sanatmetal Orthopaedic & Traumatologic Equipment Manufacturer Ltd., Hungary) used in this study harbored three proximal and four distal locking possibilities, and the most distal hole was 5 mm from the nail tip, a 15° Herzog curvature was present on the proximal side, and a 3° bend was present on the distal side for simpler introduction.

A computer-generated stratified block-randomized number series classified by the OTA stratification was used to determine treatment allocation. A single senior trauma surgeon conducted or supervised all surgeries. Fibula fixation and supplementary reduction techniques including blocking screws, percutaneous clamps, and temporary plating were used, based on the surgeon’s preference. Owing to our study design, double blinding was not possible. Data processing, statistical analyses, and assessments were conducted by staff who were unaware of the treatment assignments.

### Surgical technique

**Fig. 1:**
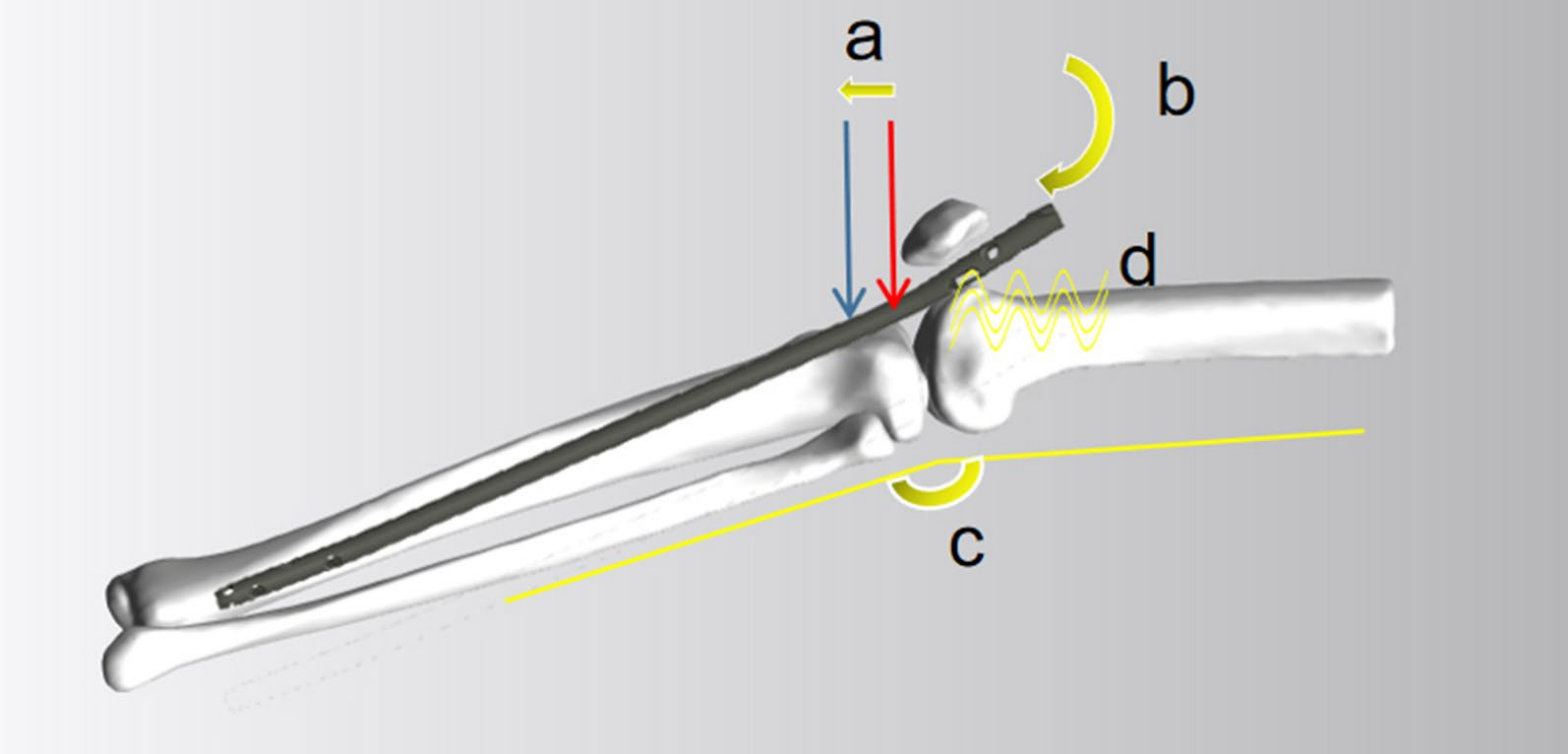
The primary unique elements of the SEIP technique include the following: *a* shows a more distal tibial entry point (the* red arrow* represents a traditional HFIP entry point while the* blue arrow* represents a SEIP entry point); *b* represents modern IMN designs, including suitable Herzog curvature on the proximal side, curvature on the distal side, and a short proximal jig; *c* shows an extra-articular approach with the knees flexed about 30°; and *d* indicates the use of a protective soft pad on the femoral side. *SEIP* semi-extended infrapatellar, *HFIP* hyperflexed infrapatellar

The HFIP technique was carried out with the patellar-tendon-split approach while placing the knee in 90° flexion. The SEIP technique was detailed in a previously published paper [13]. The primary unique elements of the SEIP technique include the following (Fig. 1): (a) a more distal tibial entry point; (b) modern IMN designs, including suitable Herzog curvature on the proximal side, curvature on the distal side, and a short proximal jig; (c) an extra-articular approach with the knees flexed approximately 30°, and (d) the use of a protective soft pad on the femoral side.

**Fig. 2:**
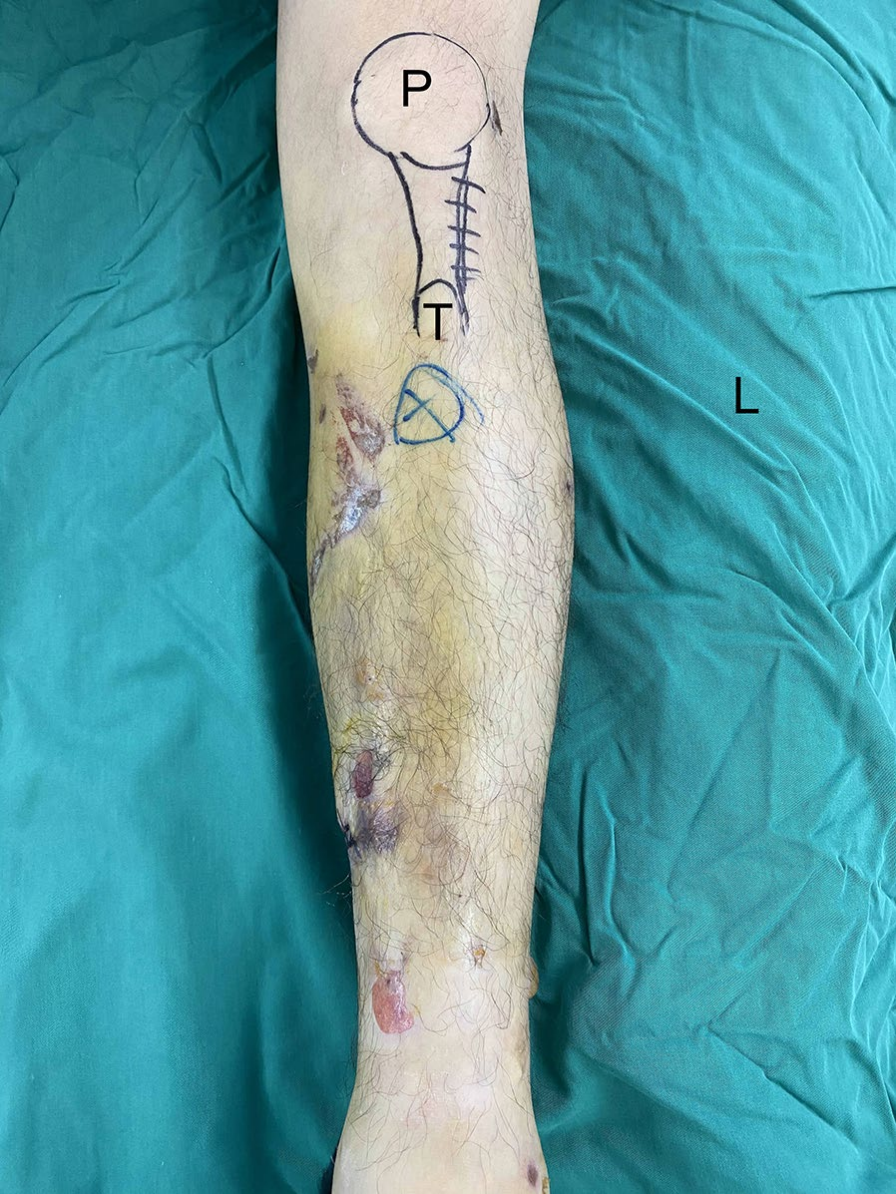
Skin incision during the SEIP technique. *SEIP* semi-extended infrapatellar, *P* patella, *T* tibial tuberosity, *L* lateral

**Fig. 3:**
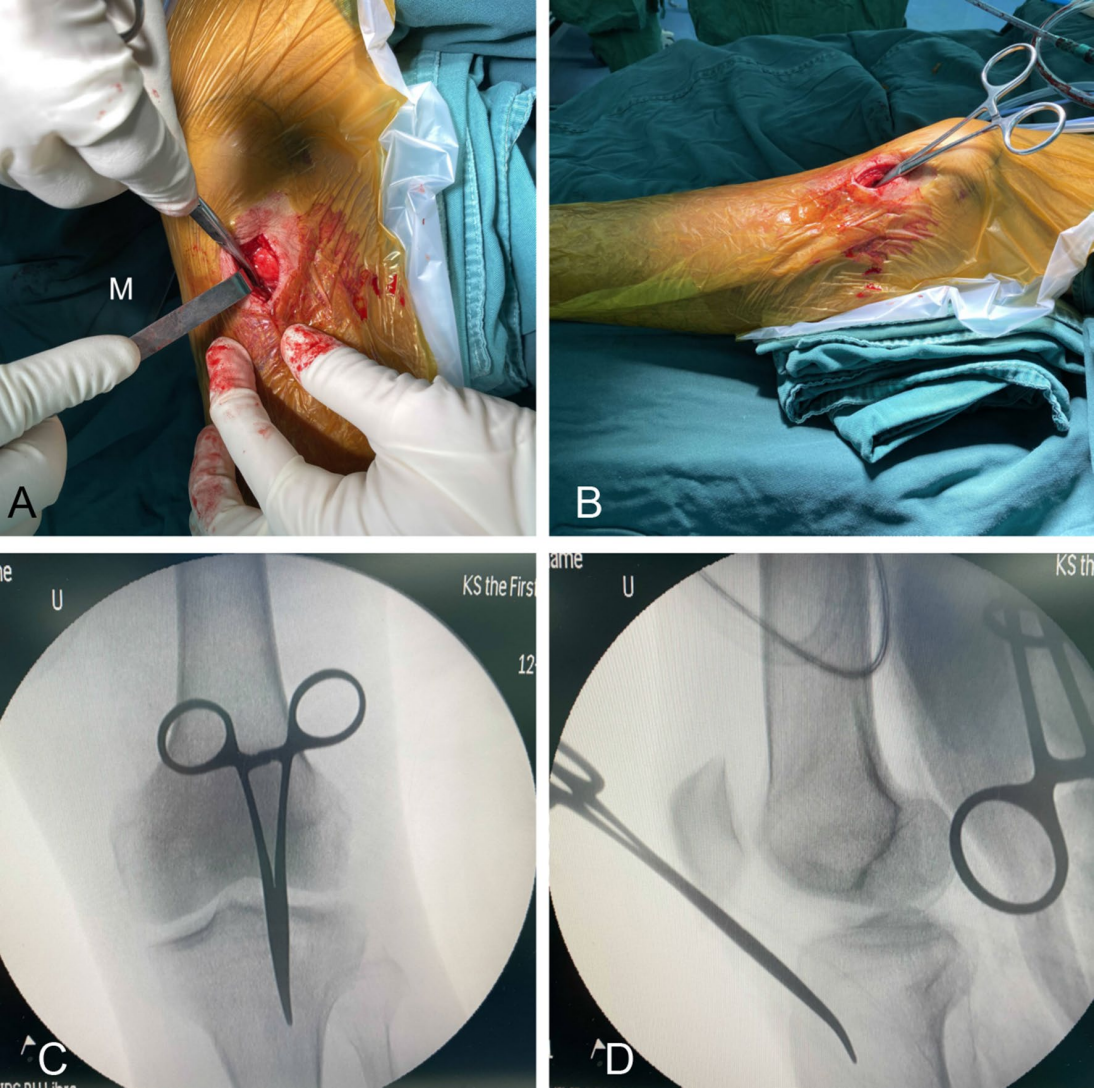
The entry point of the SEIP technique. Intraoperative photographs **A** and **B** show that the patellar tendon is pulled medially to visualize the tibial tuberosity slope. Hemostatic forceps are employed to position the tibial entry point. An ideal entry point was observed on the AP fluoroscopic view **C** as being located just medial to the lateral tibial spine, and it aligns with the tibial shaft. A lateral view **D** of the entry point located at the tibial tuberosity slope, approximately 10 mm distal to the anterior articular margin. *SEIP* semi-extended infrapatellar, *M* medial, *AP* anteroposterior

**Fig. 4:**
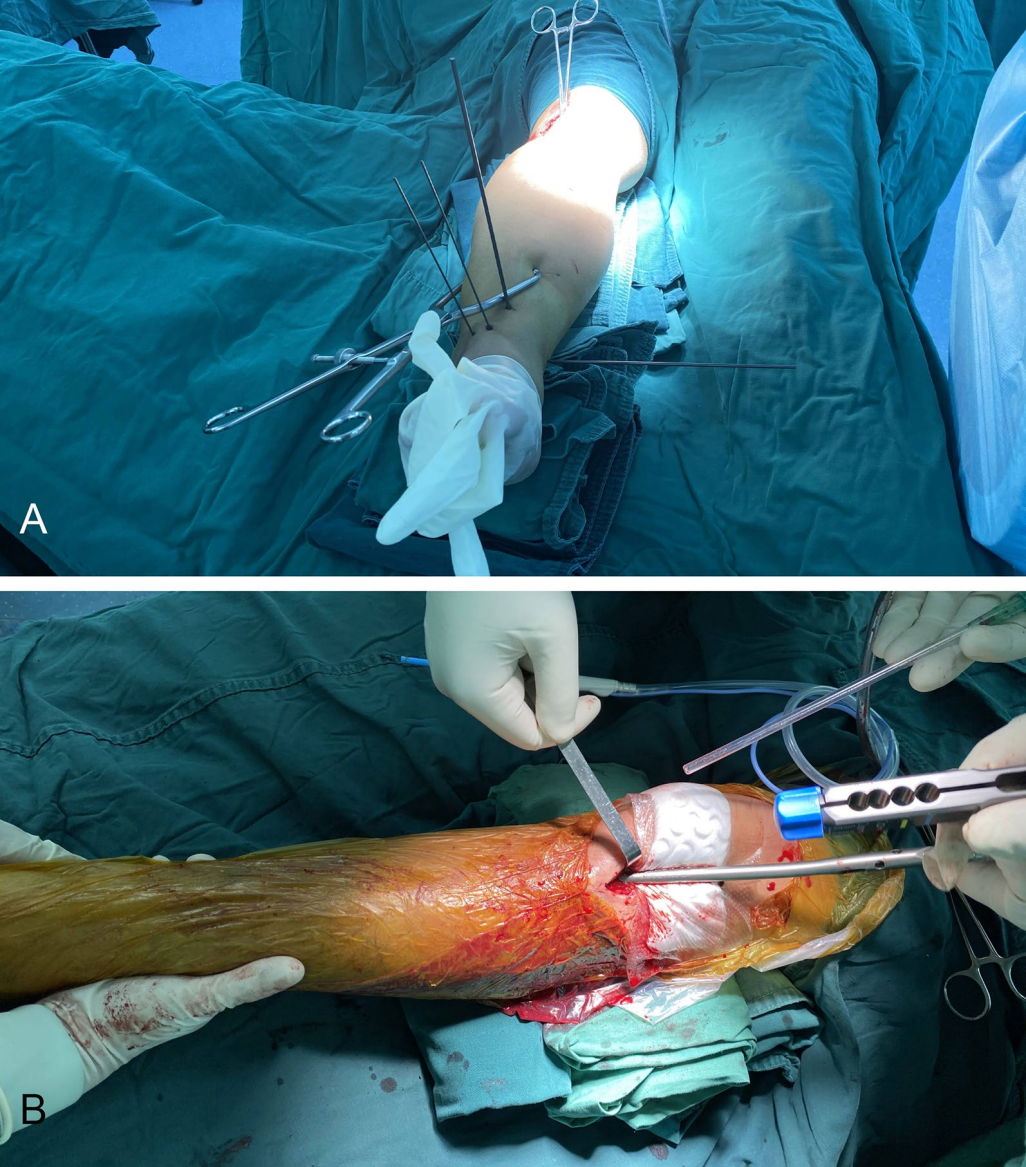
Using the SEIP technique, the distal tibial fracture reduction was carried out under the condition of almost straight lower limbs, facilitating tibial stabilization, percutaneous fixation of distal tibial fractures, fibular fixation, supplementary reduction techniques, and imaging (**A**), and there was no need to alter the position during nail insertion (**B**). Note that in the intraoperative photograph **A**, the C-arm intensifier was positioned parallel to the operating table to obtain lateral views. For AP viewing, the C-arm image intensifier was simply rotated directly above the operating table. X-ray images of the tibia segments at varying distances were obtained by simply moving the C-arm horizontally, which simplified the entire process. *SEIP* semi-extended infrapatellar, *AP* anteroposterior

In brief, the patient was laid supine on a radiolucent table with the affected leg flexed ~ 30° using a roll under the knee joint. An incision was made ~ 4–5 cm lateral to the patellar tendon (Fig. 2). Next, the patellar tendon was medially pulled to visualize the tibial tuberosity slope. Hemostatic forceps were used to position the tibial entry point, which was then confirmed using intraoperative fluoroscopy (Fig. 3A, B). The ideal entry point seen on the anteroposterior (AP) view was immediately medial to the lateral tibial spine, with perfect alignment with the tibial shaft. On the lateral view, the entry point was at the tibial tuberosity slope ~ 10 mm distal to the anterior articular margin (Fig. 3C, D). Once the entry point was identified, reduction, reaming, nail insertion, and locking were performed similar to the traditional HFIP technique except that the injured leg remained in the semi-extended position at all times (Fig. 4). In order to prevent intraoperative compression of the proximal IMN jig on the skin, a compression protective pad was placed on the femoral side.

### Post-surgical management and follow-ups

The post-surgical management and follow-ups were the same for both cohorts. For close fractures, first-generation cephalosporin was intravenously injected pre-operation and 24 h post-operation. For the Gustilo type I open fracture, the antibiotic usage duration was appropriately extended. The knee and ankle ranges of motion were supported. Moreover, the quadriceps were gradually strengthened using physical therapy. Weightbearing advancements were typically performed prior to the complete unification of bone fractures. The patients were clinically and radiologically followed up once every four weeks until union occurred. Lastly, additional follow-ups occurred at 3, 6, and 12 months after surgery.

### Outcomes

**Fig. 5:**
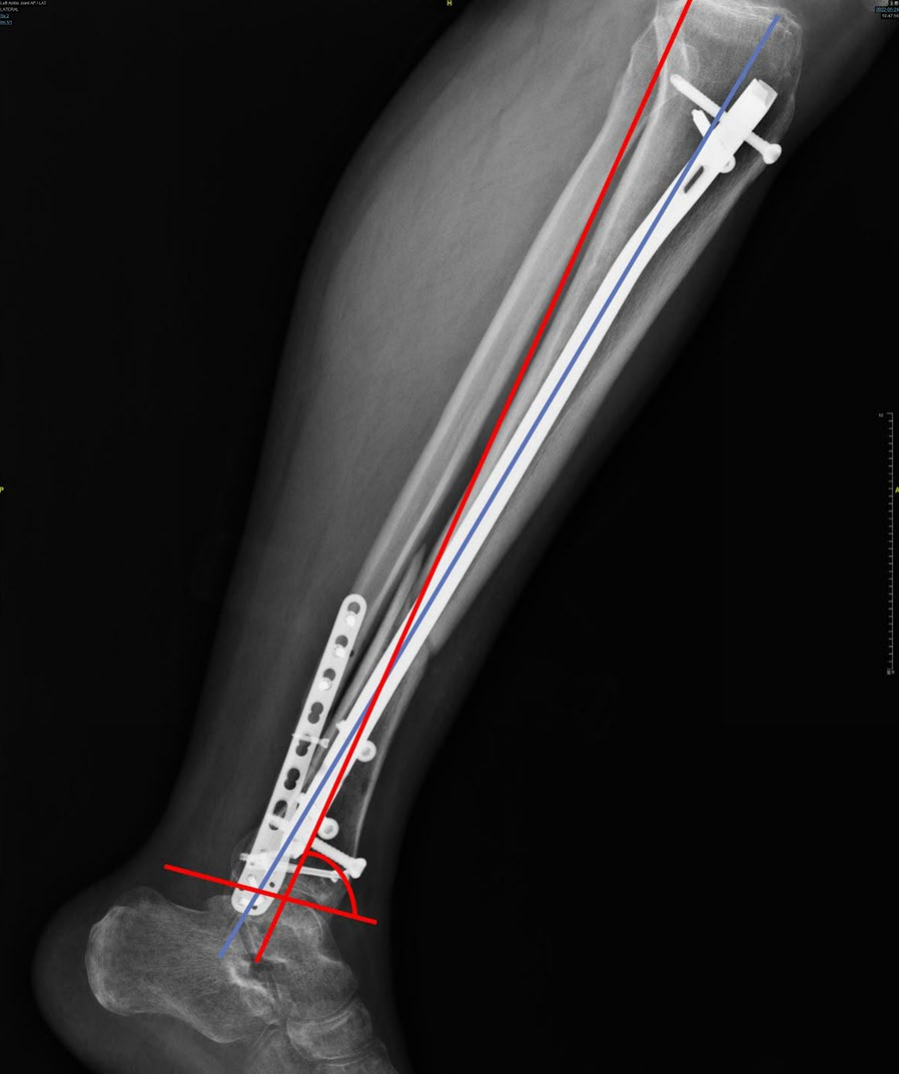
A case of malalignment following IMN: lateral view, demonstrating the difference between the proximal segment (*blue*) and distal segment (*red*) axes

The primary outcome for this study was malalignment. At each follow-up, coronal and sagittal plane radiographs of the entire knee, tibia, and ankle were obtained. Postsurgical AP and lateral tibial radiographs were assessed via the Paley technique for deformity evaluation [15]. Satisfactory radiographic alignment was described as < 5° in either the coronal or sagittal plane [8]. Figure 5 illustrates a case of malalignment following IMN. All measurements were taken by a trained senior radiologist who was unaware of the treatment assignments.

We prespecified several secondary outcomes, including intraoperative fluoroscopy time, operation time, blood loss, hospital length of stay, the functional ankle score, and complications. If the fracture healing duration was between 6 and 9 months, then it was deemed as delayed union [16]. Nonunion was a failure to unite the fracture by the ninth month post operation [17]. The functional ankle score was assessed via the American Orthopaedic Foot and Ankle Society (AOFAS) Ankle-Hindfoot Scale score [16]. To restrict bias in all clinical and manual evaluations, the last follow-up, performed at 1 year post operation, was conducted by an independent physician not involved in treating the study participants.

### Statistics

The data distribution was assessed via the Kolmogorov–Smirnoff test. Normally distributed continuous data are presented as mean (standard deviation [SD]), whilst the rest are provided as median (interquartile range [IQR]). Normally distributed data were evaluated via an independent two-tailed *t*-test. Non-normally distributed data were assessed via the Mann–Whitney U test. To assess categorical information, provided as frequency (%), the chi-squared test was employed. If the chi-squared test assumptions were violated, a Fisher’s exact test was employed instead. Multivariate analysis was performed for malalignment (binary data) information, which yielded the predicted adjusted risk ratio (RR) and 95% confidence interval (CI). The adjusted confounding factors were as follows: age, gender, fracture type, and OTA stratification. Two-sided *P* values < 0.05 were deemed as significant. The R package (http://www.R-project.org, R Foundation) was employed for all data analyses.

### Sample size

Our definition of the primary outcome (malalignment rate) was based on our preliminary examinations and a prior report [6, 18], and it was predicted to be 0.04 in the treated population (SEIP) and 0.25 in the control population (HFIP). The ratio of the number of participants in both study populations was 1:1. With an *α* value of 0.1 (one tail) and a *β* value of 0.2 (power of 0.80) in the G Power statistical analysis program, version 3.1, our patient size was determined to be 40 patients per group.

## Results

**Fig. 6:**
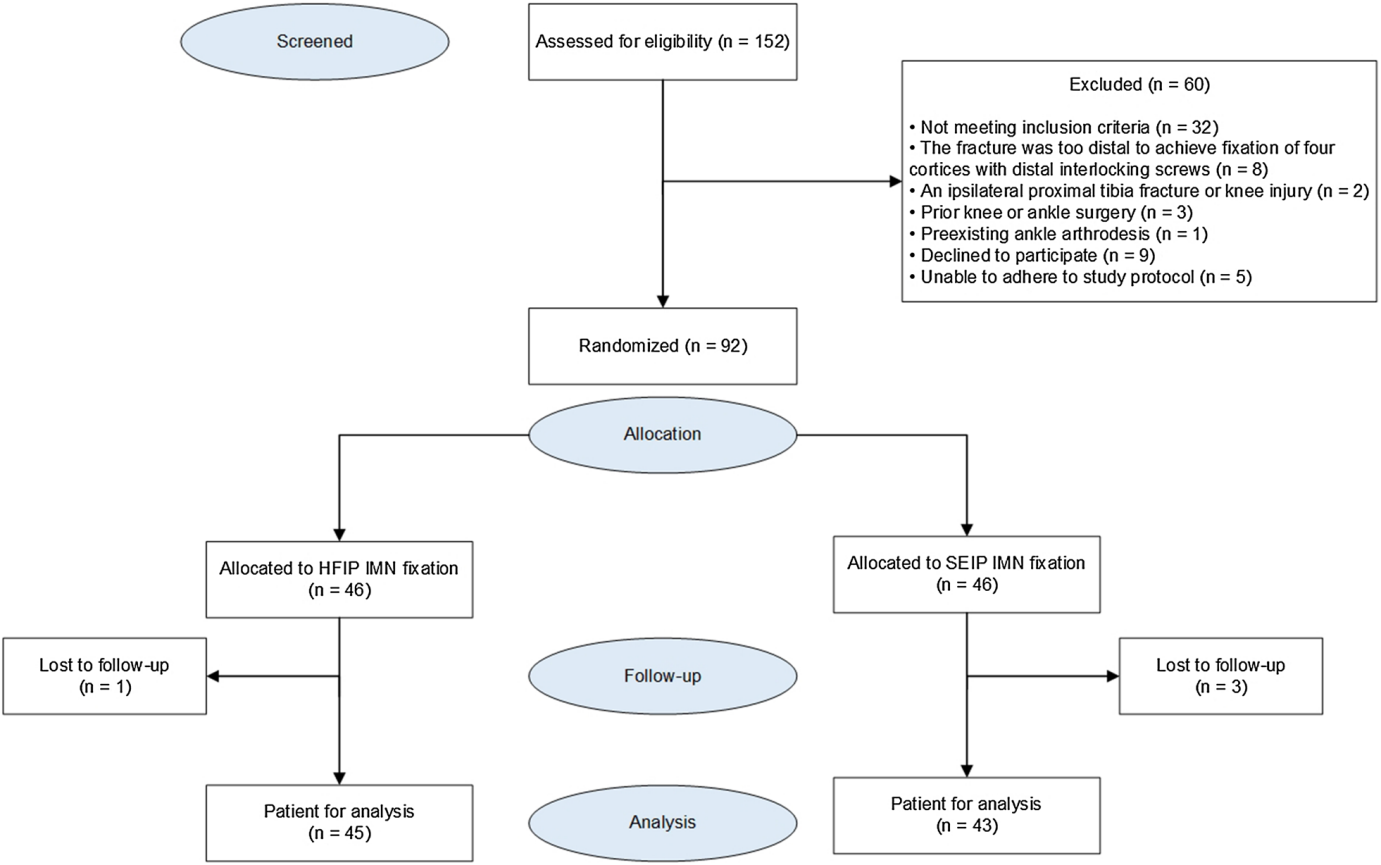
A flow diagram. *SEIP* semi-extended infrapatellar, *HFIP* hyperflexed infrapatellar, *IMN* intramedullary nail

Among the initial selection of 152 patients, only 92 were arbitrarily placed in the two study cohorts. Out of the 92 patients, 88 (96%) were present for the final 1-yr follow-up; thus, ultimately, 88 patients were used for analysis (Fig. 6). Out of the 88 patients, 45 (51%) underwent traditional HFIP IMN and 43 (49%) underwent SEIP IMN. They had an average (SD) age of 36.8 (9.2) years, and 41% were female. Both cohorts showed comparable baseline data (*P* values > 0.05), as presented in Table 1. As detailed in Table 2, we observed no difference in the fibular fixation (*P* value = 0.360) or supplementary reduction technique (*P* values > 0.05) rates during the fixations in both cohorts.

**Table 1:** Patient baseline characteristics

Characteristics	HFIP (*n* = 45)	SEIP (*n* = 43)	Standardized diff. (95% CI)	*P* value
Age, mean (SD), yrs	37.3 ± 8.8	36.3 ± 9.6	0.1 (− 0.3, 0.5)	0.624
Gender, *n* (%)			0.1 (− 0.4, 0.5)	0.798
Male	26 (57.8%)	26 (60.5%)		
Female	19 (42.2%)	17 (39.5%)		
Type of fracture, *n* (%)			0.1 (− 0.3, 0.5)	0.542
Close	34 (75.6%)	30 (69.8%)		
Open (Gustilo type I)	11 (24.4%)	13 (30.2%)		
OTA classification, *n* (%)			0.1 (− 0.3, 0.6)	0.791
43-A	38 (84.4%)	34 (79.1%)		
43-C1	5 (11.1%)	6 (14.0%)		
43-C2	2 (4.4%)	3 (7.0%)		

*HFIP* hyperflexed infrapatellar, *SEIP* semi-extended infrapatellar, *SD* standard deviation, *CI* confidence interval, *OTA* Orthopaedic Trauma Association

**Table 2:** Fibula fixation and supplementary reduction techniques used during fixation

	HFIP (*n* = 45)	SEIP (*n* = 43)	*P* value
Fibular fixation, *n* (%)	20 (44.4%)	15 (34.9%)	0.360
Supplementary reduction techniques			
Blocking screws, *n* (%)	8 (17.8%)	7 (16.3%)	0.852
Percutaneous clamps, *n* (%)	12 (26.7%)	11 (25.6%)	0.908
Plate for provisional fixation, *n* (%)	5 (11.1%)	4 (9.3%)	0.780

*SEIP* semi-extended infrapatellar, *HFIP* hyperflexed infrapatellar, *SD* standard deviation

### Primary outcome

**Fig. 7:**
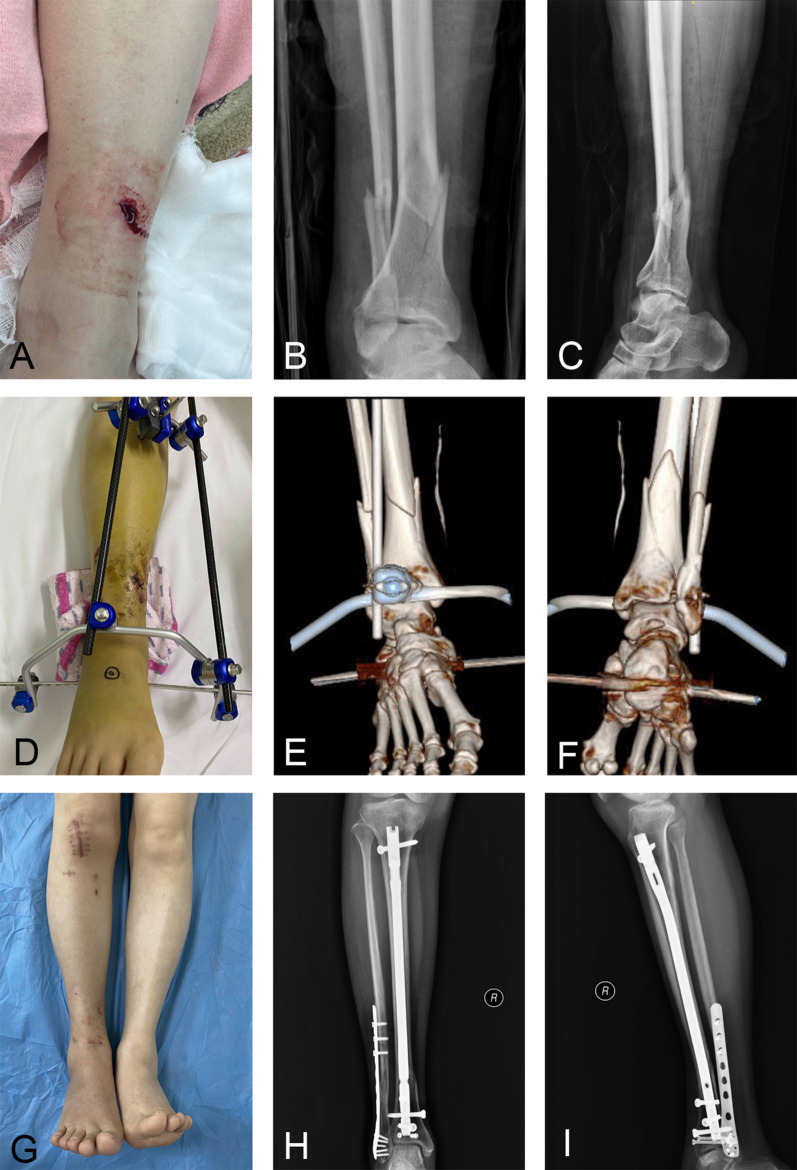
A 57-year-old woman sustained an open distal tibia and fibular fracture. **A**, **B**, and **C** illustrate that the tibial fracture was Gustilo type I, OTA 43-C1 DTF. **D** reveals that the patient underwent closed reduction and external fixation. When using three-dimensional computed tomography, a nondisplaced intraarticular fracture line was obviously visible (**E** and **F**). **G** depicts the overall image at the 3-month follow-up after SEIP IMN surgery. **H** and **I** depict the AP and lateral radiographs of the tibia at the 8-month postoperative follow-up. *SEIP* semi-extended infrapatellar, *IMN* intramedullary nail, *AP* anteroposterior, *DTF* distal tibial fracture

Table 3 summarizes the outcomes of this study. Malalignment occurred in 9 patients (20.0%) among the HFIP population and in 2 patients (4.7%) among the SEIP population (*P* value = 0.030). The adjusted RR among SEIP versus HFIP patients was 0.16 (95% CI 0.00 to 0.58). Given this evidence, the SEIP technique markedly reduced the incidence of malalignment compared to the HFIP technique, with an average reduction of 84% among the SEIP patients. Figure 7 depicts a typical case who underwent SEIP IMN fixation. In this patient, there was no evidence of postoperative malalignment.

**Table 3:** Outcomes of the study

Outcomes	HFIP (*n* = 45)	SEIP (*n* = 43)	*P*-value	Adjusted RR (95% CI)^b^
Primary outcome				
Malalignment, *n* (%)	9 (20.0%)	2 (4.7%)	0.030	0.16 (0.00 to 0.58)
Secondary outcomes				
Fluoroscopy time, mean (SD), min	18.7 (4.4)	14.9 (2.9)	< 0.001	
Operation time, mean (SD), min	123.6 (31.9)	105.6 (17.4)	0.002	
Blood loss, mean (SD), mL	161.3 (87.7)	140.7 (83.7)	0.263	
Hospital length of stay, mean (SD), d	11.7 (2.4)	11.6 (2.7)	0.878	
AOFAS Ankle-Hindfoot Scale score^a^, mean (SD)	84.3 (8.6)	88.5 (5.8)	0.008	
Complications				
Superficial infection, *n* (%)	6 (13.3%)	7 (16.3%)	0.697	
Deep infection, *n* (%)	0 (0)	0 (0)	NA	
Delayed union, *n* (%)	5 (11.1%)	4 (9.3%)	0.780	
Nonunion, *n* (%)	2 (4.4%)	1 (2.3%)	0.584	

*SEIP* semi-extended infrapatellar, *HFIP* hyperflexed infrapatellar, *CI* confidence interval, *SD* standard deviation, *AOFAS* American Orthopaedic Foot and Ankle Society, *RR* risk ratio, *OTA* Orthopaedic Trauma Association

^a^Range, 0 to 100; higher scores indicate better function

^b^Risk ratios were adjusted for age, gender, type of fracture (close or open), and OTA classification

### Secondary outcomes

In terms of the secondary outcomes (Table 3), the mean intraoperative fluoroscopy duration among the SEIP population was 14.9 min (SD, 2.9 min), which was significantly less (*P* value < 0.001) than the standard HFIP technique (mean, 18.7 min; SD, 4.4 min). Similarly, the surgical duration was much smaller in the SEIP versus HFIP patients (mean, 105.6 min; SD, 17.4 min; vs. mean, 123.6 min; SD, 31.9 min; *P* value = 0.002). The AOFAS Ankle-Hindfoot Scale score of the SEIP patients was significantly elevated compared to the HFIP patients (mean, 88.5; SD, 5.8; vs. mean, 84.3; SD, 8.6; *P* value = 0.008). However, the intraoperative blood loss and hospitalization duration showed no significant difference between the two study populations (*P* values > 0.05).

### Complications

Superficial infections were detected and treated via dressing alteration and antimicrobial therapy in 13 patients. None of these patients developed deep infections, which required additional procedures. The superficial and deep infection rate showed no significant difference between the two study populations (*P* values > 0.05). Delayed union was detected in 4 SEIP patients (9.3%) and in 5 HFIP patients (11.1%). The delayed union rate showed no significant difference between the two cohorts (*P* value = 0.780). In total, 7 of 9 patients were treated with nail dynamization, among which 6 healed uneventfully. However, 1 SEIP patient (2.3%) and 2 HFIP patients (4.4%) developed nonunion. We also observed no marked difference in nonunion rate between the two groups (*P* value = 0.584). Among the 3 nonunion patients, 2 patients received exchange nailing and iliac crest bone grafting, and 1 underwent nail removal, compression plating, and iliac crest bone grafting. Subsequently, these patients achieved complete tibial fracture healing. Notably, the nail endcap protruded in 1 patient (2.3%) in the SEIP group; however, there was no observable irritation. Hence, there was no statistically significant difference in device prominence and irritation (*P* values > 0.99).

## Discussion

This RCT involving 88 DTF patients demonstrated a substantially reduced malalignment rate when using the SEIP IMN technique compared to the traditional HFIP IMN technique. In addition, the SEIP technique minimized the intraoperative fluoroscopy time and operation time and improved the postoperative ankle function. We are the first to report a marked impact of the IP nail insertion technique while treating an extraarticular or a nondisplaced intra-articular DTF. This prospective study extends the indications for this novel SEIP technique from tibial shaft fracture to DTF.

Currently, the primary interventions for treating DTF are IMN, locked plate, and external fixation [19, 20]. The usage of the above surgical procedures depends on the fracture site, related fracture, ankle joint component, soft-tissue status, and patient’s general health. Prior investigations suggested that different DTF fixation methods elicit differing complication patterns. In particular, infections, wound complications, and implant prominence are widely reported following tibial plating, whereas malalignment and knee pain are correlated with IMN [7, 16, 21–24]. The precise reduction of the distal fragment minimizes the risk of tibiotalar joint malalignment, which contributes to the uneven loading of joint surfaces [25]. Multiple studies have demonstrated that angular malalignment contributes to adverse long-term functional outcomes. Moreover, it was shown that a malalignment of only 5° can induce substantial ankle/knee pain [26] and subtalar joint stiffness [27]. Malalignment may be linked to osteoarthritis; however, there are no reports of a statistical correlation between the two [27]. Thus, when choosing IMN for DTFS treatment, a precise reduction and alignment and the maintenance of the alignment is crucial for treatment success. However, the traditional HFIP technique requires the knee to be flexed  > 90°. To enable nail entry, the knee is generally hyperflexed while resting on a support. This hyperflexed position poses certain challenges due to the considerable leg manipulation that is required for fluoroscopic intraoperative visualization. Eccentric reaming or a failure to control the distal segment may therefore cause substantial malalignment and limb deformity [8].

To address the challenges of the hyperflexed position, several IMN approaches have been proposed which employ the semi-extended position. These approaches are the PP approach, which utilizes either an extra- or intra-articular nail insertion either lateral or medial to the patellar tendon [28–30], and the SP approach, which allows nail insertion to pass through the quadriceps tendon [31]. Several studies have revealed that the SP IMN technique produces a markedly reduced malalignment rate relative to the HFIP IMN technique when treating DTFs. In a retrospective investigation of 266 patients, Avilucea et al. observed that a primary angular malalignment of  > 5° was present in 35 (26.1%) patients with HFIP IMN insertion, and in 5 (3.8%) patients with SP IMN insertion [8]. In another retrospective cohort study comparing 74 SP and 51 HFIP nails, Hague et al. suggested that the SP technique may enhance coronal plane alignment with the intramedullary nailing of DTFs [32]. Lu et al. analyzed and compared the clinical and functional endpoints of DTFs managed with IMN, and the authors reported that the malalignment rate in SP patients (4.8%, 2/42) was considerably diminished compared to HFIP patients (14.3%,8/56) [14]. In the current study, the malalignment rate of SEIP patients was 4.7%, which was markedly reduced relative to HFIP patients, which corroborated the radiographical outcomes of a prior investigation. In addition, we employed the AOFAS Ankle-Hindfoot Scale score to assess the functional outcomes. Our results were also consistent with a study by Lu et al. which compared the postoperative AOFAS scores of DTFs treated using the HFIP and SP approaches [14]. This may be because of the reduced malunion rate of DTFs in the semi-extended position compared to the hyperflexed position. It is our belief that the same low malalignment rate and good function are present when using either SEIP IMN or SP IMN to treat DTFS. However, tio date, there is no clinical evidence to support this hypothesis, and so prospective clinical studies are warranted to compare the SEIP and SP approaches in the future.

The SEIP-technique-related low malalignment rate during DTF treatment may also be due to the intrinsic feature of the semi-extended position. The precise management of the leg and distal fracture segment is critical for operation success. The semi-extended position allows the tibia to be in a resting state on a horizontal surface through the entirety of the surgical procedure, which facilitates the tibial stabilization and imaging [29]. Moreover, if the metaphyseal area is not able to provide sufficient stability or if proper reduction is not achieved, then blocking screws are needed to constrict the metaphyseal canal and guide the nail and fragment. The blocking screws procedure requires X-ray guidance, and this becomes challenging or impossible in the AP direction owing to the hyperflexion position of the patient leg. However, the SEIP technique, like other semi-extended techniques, resolves this concern when treating DTFs, thus making the blocking screws procedure easier, faster, and more secure. Similarly, the SEIP technique provides similar advantages in terms of distal locking, percutaneous fixation of DTFs, fibular fixation, percutaneous clamps, and temporary plating.

Despite reports of a reduced risk of intra-articular seeding during SP nailing of an open tibia fracture [33, 34], the possibility of grossly contaminated open fractures is concerning. Therefore, one must monitor the small, but present, risk of septic arthritis [34]. Relative to the conventional semi-extended and HFIP techniques, the surgical site for the SEIP approach is located further away from the knee joint. In theory, the SEIP approach may further minimize knee sepsis risk in high-risk patients such as those with open tibial fractures. Likewise, the extra-articular feature of the SEIP approach may address surgeons' concerns regarding iatrogenic damage originating from either canal preparation, nail insertion, or enhanced contact pressure on the patella and femoral condyles [35]. In particular, in some Asian populations, the patellofemoral joint space is much smaller than in westerners. Therefore, the SP approach with instrumentation usage in such a narrow space may induce further damage [10, 36]. Thus, in terms of short individuals, the SEIP approach may offer an extra-articular alternative requiring no special surgical instruments. Likewise, the SEIP approach may also be suitable for severe osteoarthritis patients with limited patellofemoral joint space.

Kubiak et al. previously developed a semi-extended extra-articular PP approach [29]. The corresponding modified techniques have since been published [28, 37]. Small capsular rupture is, however, possible, and would require extra repair [29]. Additionally, there is a chance of postoperative patellar instability brought on by retinacular repair failure [29]. The most significant technical difference between the SEIP and the prior extra-articular PP approaches is a more distant tibial entry point, which may reduce the chance of injuring the retinaculum, surrounding tissue, and joint capsule. Further studies are warranted to confirm this hypothesis in the future.

With a rising number of patients who receive or will receive total knee arthroplasty (TKA), the SEIP method is critical for tibial nailing, particularly to avoid intra-articular penetration. Devendra et al. demonstrated a successful modified HFIP IMN approach in a case study involving 3 patients who presented with tibial shaft fractures distal to the location of TKA prostheses [38]. Similarly, Haller et al. reported using an IMN with a modified HFIP procedure to correct DTF, whereby they successfully avoided the tibial baseplate and thus provided stable fracture fixation, which facilitated early weight bearing [39]. The primary technique used in the aforementioned two studies is a more distal patellar-tendon-split approach, which coincides with our SEIP technique. Thus, we suggest that the SEIP procedure can be utilized for this fracture pattern distal to a TKA, particularly if there is adequate space to accommodate the nail and instrumentation proximal and anterior to the tibial tray.

The SEIP technique can also be applied to the treatment of tibial fractures associated with proximal tibial plateau fractures. The SEIP approach can be extended proximally and distally, converting it into an open access in relation to the articular surface after a sub-meniscal arthrotomy. Owing to a more distal tibial entry point, the tibial plateau articular surface can be first reduced and temporarily fixed. Following nailing, the proximal tibia should have sufficient space for subchondral raft screws combined with the locking plate fixation.

Our research has certain limitations that need to be addressed. Firstly, it is a single-center investigation involving a relatively small sample population and a short-term follow up. Hence, a large-scale, multicenter RCT is warranted to assess the efficacy of the SEIP procedure. Secondly, only one type of IMN was employed in this study, and the efficacies of other IMN types are unknown. Thirdly, since it was not possible to make this a double-blinded study, surgeons were aware of the employed procedures and may have been more careful while applying novel procedures. Fourthly, the conventional semi-extended approaches (SP or PP) were not designed for comparison. Finally, rotational malalignment is a potential complication following IMN [40, 41]. However, we did not include this important measure as an end point. Further studies involving this end point can be carried out in the future.

## Conclusions

In summary, we demonstrated that the SEIP IMN technique markedly enhances extraarticular and nondisplaced intra-articular DTFs alignment relative to the traditional HFIP IMN technique. The described technique represents an effective option for IMN of DTFs.

## Data Availability

The datasets used during the current study are available from the corresponding author on reasonable request.

## Abbreviations

RCT: Randomized clinical trial
HFIP: Hyperflexed infrapatellar
SEIP: Semi-extended infrapatellar
IP: Infrapatellar
PP: Parapatellar
SP: Suprapatellar
MIPO: Minimally invasive plating
IMN: Intramedullary nailing
DTF: Distal tibia fracture
AOFAS: American Orthopaedic Foot and Ankle Society
OTA: Orthopaedic Trauma Association
TWX-E: Tibia Without X-ray-Excellent
AP: Anteroposterior
RR: Risk ratio
SD: Standard deviation
IQR: Interquartile range
CI: Confidence interval
TKA: Total knee arthroplasty
